# Research in the Training of Cardiovascular Surgeon

**DOI:** 10.21470/1678-9741-2021-0612

**Published:** 2022

**Authors:** Andy Petroianu

**Affiliations:** 1 Department of Surgery, Faculdade de Medicina, Universidade Federal de Minas Gerais, Minas Gerais, Brazil

I gratefully acknowledge Professor Dr. Paulo Roberto Barbosa Evora for the honorable invitation to present some reflections on research in the training of cardiovascular surgeons. The ephemerality that characterizes the scientific truths, as new information substitutes the provisionally established knowledge, is also valid for this writing^[1-4]^. In the past, I had other concepts, and the future will make me have a different view on this topic.

Science is a part of Philosophy, as it is a style of thought and action, which seeks to solve problems arising in human activities or originating in thought and uses the research as an instrument that, by being creative, is an Art. Medicine is the human activity whose character associates Science with the Art of Research, thus physicians should be considered researchers in their activity^[1-8]^. Like Molière’s Bourgeois, who spent his life making “prose” without realize it, all physicians carry out research throughout their activity without being aware of this fact^[9]^. Therefore, it makes no sense for the cardiovascular surgeons to reject the research, considering themselves unable to exercise it or alleging lack of time, for being far from their purposes. Physicians, regardless of their specialty, act as researchers by accepting, directly or indirectly, the challenge of each patient, in search of the correct diagnosis and the best therapeutic result. Persistence in their objectives qualifies researchers and physicians alike, in laboratories, advanced technological institutes, offices, or the operating room^[8,10,11]^.

“Know thyself” (γνῶθι σɛα&ugr;τόν), found in the temple of Apollo at Delphi and written about 3500 years ago, contains the essentials of making choices, including the professional ones^[7]^. All people are born genetically made, not only in physical and functional characteristics, but intellectually too^[3,8,12]^. True surgeons already have innate talent and skill for the surgical art, and to choose the specialty with which each one best identifies is a personal decision. The Cardiovascular Surgery is much more than limited to the heart and great vessels, as it can be verified in many thousands of books and millions of scientific papers. This is one of the Medicine fields in which most research is done, both in the basic sciences, in laboratories and with animals, as in the clinic and surgical practice.

No matter how much it is studied, researched, and published, there will always be many mysteries in all fields of knowledge to be investigated and understood^[2,3,5]^. Every day, in each patient, doubts and new findings must be disclosed, despite the vast literature^[13]^.

In other countries, with advanced social awareness, scientific works are stimulated by substantial financial resources from government programs and many organizations and institutions that sponsor research projects. Specifically in Medicine, grants not only for the researchers but also for the hospitals and laboratories where they work motivate physicians to produce, to not being replaced by other more productive professionals. Thus, the surgeon must associate surgical excellence with the qualities of a fecund researcher. As a result, there is a personal financial gain combined with the spread of the professional name at a high intellectual and social level in a continuous upward spiral.

In Brazil, research funding is almost exclusively provided by public institutions, with emphasis on the Conselho Nacional de Desenvolvimento Científico e Tecnológico (CNPq), Coordenação de Aperfeiçoamento de Pessoal de Nível Superior (CAPES), and states Fundações de Apoio à Pesquisa (FAPs)^[8,14,15]^. Regrettably, the current government policy has progressively reduced resources, considering Brazilian research to be superfluous and preferring to import the knowledge generated by other countries for being less expensive. On the other hand, there is no incentive for private institutions to sponsor the research. Traditionally, Brazilian and multinational companies prefer to stimulate physicians to consume their products instead of investing in professionals to contribute to the development of drugs, devices, instruments, and equipment, which would certainly be useful to the enterprises themselves and to promote the national scientific progress.

In the cardiovascular area, there is also the financial income proportional to the surgical activity, therefore, the more the surgeon operates, the greater will be the gain. In this way of thinking, research is seen as a waste of time and, consequently, of money. Scientific investigation is laborious, requires the preparation of a project, which must be submitted to an ethics committee and only after its approval, which is not easy, the work is allowed to be carried out. It is also up to the researcher to seek financial support to implement the research, considering that the reduced resources from the funding agencies are directed to the most productive researchers, as they are more likely to conclude and publish their experiments.

In this sad scenario, when compared to other countries, research in Brazilian Cardiovascular Surgery remains at a level far below its real capacity and its professionals will. Even so, there are many skilled surgeons, with a relevant clinic, who still find time to carry out scientific works of great importance, often supported by their own resources. This reality confirms the investigative nature of all physicians, including the cardiovascular surgeons.

Despite the positive or negative results, the research must be published, as it is not possible to predict its repercussion in the medical-scientific community. Every work brings some kind of contribution. Even unsuccessfully frustrating investigations can help other researchers, preventing them from repeating the same work, which has proven inadequate^[1,4,16-18]^. It is unacceptable that an idea, a discovery during the professional practice, or a research is not published.

Publishing the manuscript in an international high impact factor journal should not be the main purpose of an author. Although an article published in a journal with a greater impact is more valued, it is not necessarily more read. The information contained in the article is most useful when directed to the community that has a real interest in it. Researchers should select the journal that best reaches this community. Currently, all articles published in the journals integrated in each database are included without considering the impact factor. Articles published in journals with a high impact factor and others with no impact are found side by side. Likewise, letters to the editor are put together with case reports, review articles, and multicenter studies, as well as advanced research, very elaborate and on the edge of knowledge. Each article is considered and cited for its intrinsic value and no longer for the vehicle that published it^[19]^.

Science helps in individual progress, however, if it is not associated with a greater humanistic development, the physician, being a researcher or not, will remain small^[2,3,7,12]^. Occasionally, a scientific work can have social repercussions and bring worthwhile returns, but the biggest reward is to overcome a challenge and to feel useful, through an honest activity and within its own limits. Desiring to contribute to society in solving problems in the most different fields of knowledge is very similar to creating a work in Art and even giving birth to a child. The immortality of the life is achieved by means of the reproductive capacity and by the products that, due to their value, persist after the owner’s departure.
